# Genomics insights into flowering and floral pattern formation: regional duplication and seasonal pattern of gene expression in *Camellia*

**DOI:** 10.1186/s12915-024-01851-y

**Published:** 2024-02-27

**Authors:** Zhikang Hu, Zhengqi Fan, Sijia Li, Minyan Wang, Mingchuan Huang, Xianjin Ma, Weixin Liu, Yupeng Wang, Yifan Yu, Yaxuan Li, Yingkun Sun, Xinlei Li, Jiyuan Li, Hengfu Yin

**Affiliations:** 1 State Key Laboratory of Tree Genetics and Breeding, Research Institute of Subtropical Forestry, Chinese Academy of Forestry, Hangzhou, 311400 Zhejiang China; 2 College of Information Science and Technology, Nanjing Forestry University, Nanjing, 210037 China; 3 Key Laboratory of Forest Genetics and Breeding, Research Institute of Subtropical Forestry, Chinese Academy of Forestry, Hangzhou, 311400 Zhejiang China; 4 College of Horticulture, Qingdao Agricultural University, Qingdao, 266109 Shandong China

**Keywords:** *Camellia japonica*, Genome, Ornamental traits, Annual gene expression, Genome duplication

## Abstract

**Background:**

The formation and domestication of ornamental traits are influenced by various aspects, such as the recognition of esthetic values and cultural traditions. *Camellia japonica* is widely appreciated and domesticated around the world mainly due to its rich variations in ornamental traits. Ornamental camellias have a diverse range of resources, including different bud variations from *Camellia* spp. as well as inter- and intra- specific hybridization. Despite research on the formation of ornamental traits, a basic understanding of their genetics and genomics is still lacking.

**Results:**

Here, we report the chromosomal-level reference genome of *C. japonica* through combining multiple DNA-sequencing technologies and obtain a high-density genetic linkage map of 4255 markers by sequencing 98 interspecific F_1_ hybrids between *C. japonica* and *C. chekiangoleosa*. We identify two whole-genome duplication events in *C. japonica*: one is a shared ancient γ event, and the other is revealed to be specific to genus *Camellia*. Based on the micro-collinearity analysis, we find large-scale segmental duplication of chromosome 8, resulting to two copies of the *AGAMOUS* loci, which may play a key role in the domestication of floral shapes. To explore the regulatory mechanisms of seasonal flowering, we have analyzed year-round gene expression patterns of *C. japonica* and *C. azalea*—a sister plant of continuous flowering that has been widely used for cross breeding. Through comparative analyses of gene co-expression networks and annual gene expression patterns, we show that annual expression rhythms of some important regulators of seasonal growth and development, including *GIGANTEA* and *CONSTANS* of the photoperiod pathway, have been disrupted in *C. azalea*. Furthermore, we reveal that the distinctive expression patterns of *FLOWERING LOCUS T* can be correlated with the seasonal activities of flowering and flushing. We demonstrate that the regulatory module involved in *GIGANTEA*, *CONSTANS*, and *FLOWERING LOCUS T* is central to achieve seasonality.

**Conclusions:**

Through the genomic and comparative genomics characterizations of ornamental *Camellia* spp., we propose that duplication of chromosomal segments as well as the establishment of gene expression patterns has played a key role in the formation of ornamental traits (e.g., flower shape, flowering time). This work provides a valuable genomic platform for understanding the molecular basis of ornamental traits.

**Supplementary Information:**

The online version contains supplementary material available at 10.1186/s12915-024-01851-y.

## Background

Camellias is known worldwide for its diverse flower patterns and floral colors, which is predominately domesticated from *Camellia japonica*. *C. japonica* belongs to genus *Camellia* and is a sister species to *C. sinensis* (tea) and *C. oleifera* (oil-tea) [1, 2]. Wild populations of *C. japonica* are found mainly in coastal areas of eastern China, Japan, and southern Korean Peninsula [3]. In China, the *C. japonica* “Naidong” (cjaND) is a local ecotype distributed in the islands of the Yellow Sea, representing the northernmost wild habitats [4]. Compared with other natural *C. japonica* populations, the wild cjaND appears to have higher genetic diversity [4, 5].

**Fig. 1:**
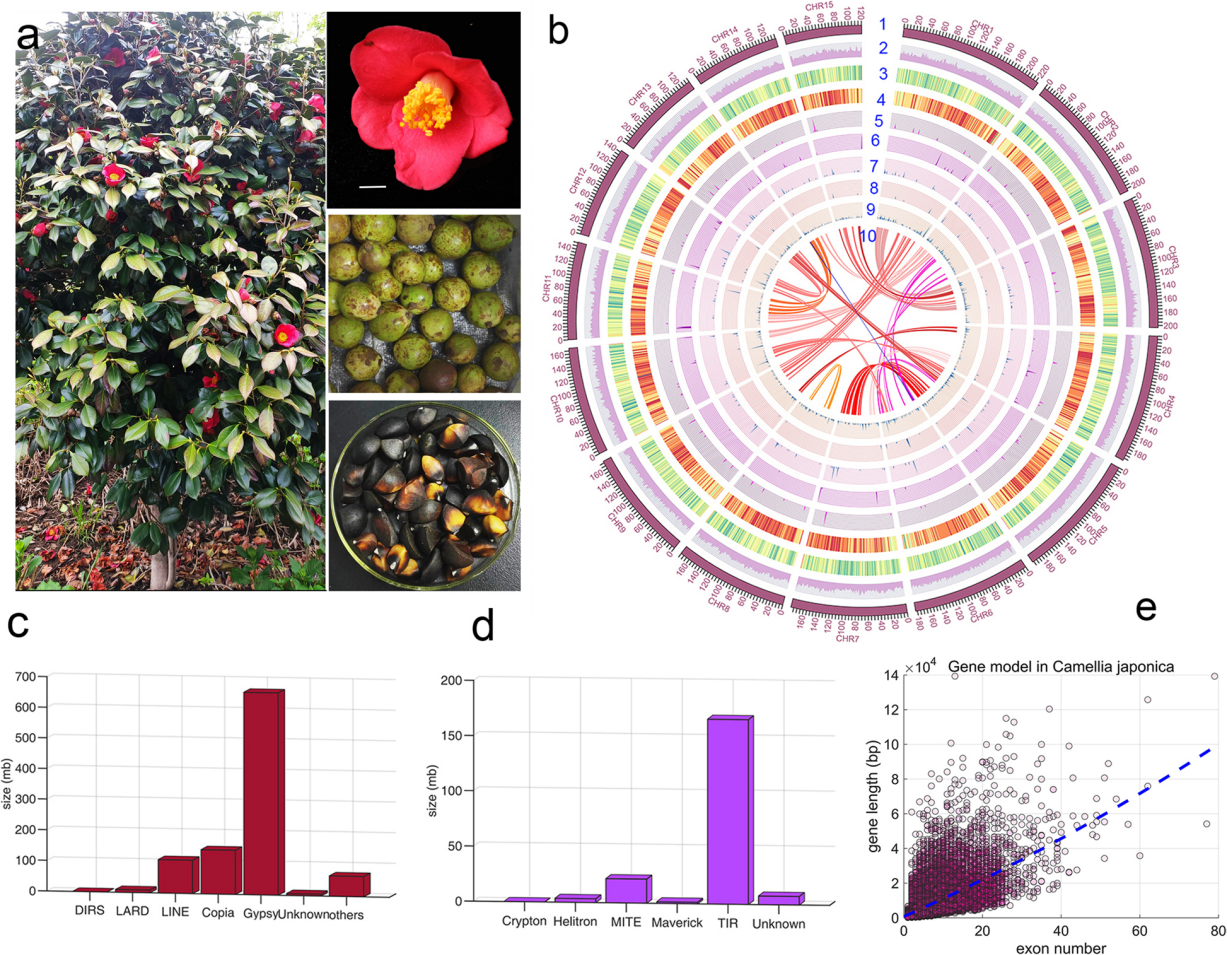
Overviews of *C. japonica* (cjaND) reference genome and construction of the genetic linkage map. **a** An overall photograph of the cjaND tree used for genome sequencing. The close-up photos of flowers, seeds, and fruits are on the right. **b** Circular representation of the assembled cjaND genome features. Different layers of circles are listed as follows: (1) the pseudo-molecules of chromosomes; (2–9) the distribution of GC, repetitive elements, gene models, noncoding RNAs, microRNAs, rRNA, snoRNA and tRNA; (10) the syntenic regions within the cjaND genome. For details of setting the Circos plot, see the supplementary files. The genome of cjaND has high levels of retrotransposons (**c**) and DNA transposons (**d**), which are classified based on the sequence similarity search. **e** Scatter plot of gene length and exon number based on the genome annotation of cjaND

The use and selection of attractive *C. japonica* plants have a long history. *C. japonica* and many close relatives that bearing unique floral morphologies or blooming seasonality are commonly used in camellias breeding [2]. Unlike most ornamental double-flowered camellias, wild *C. japonica* flowers consist of a single whorl of red petals (usually 5–7 petals) and multiple rounds of stamens, and its spherical capsules contain several oil-rich seed kernels [2] (Fig. 1a). The floral ABC model genes have been found to play a key role in the formation of double flowers in camellias [6]. Consistent with the ABC model, the weakening or elimination expression of the *AGAMOUS* homologous gene (*CjAG*) is associated with the formal doubled-flowers in *C. japonica* [7]. Unexpectedly, in anemone double flowers, the expression of *CjAG* is upregulated in the inner petals [7], indicating other regulators are involved in different types of double flowers in *C. japonica*. In terms of artificial selection of flower colors, *Camellia* varieties also have their own rich variations of petal color: for example, a range of varieties with traditional flower color variations from white to pale pink to deep red and a range of variations from white to yellow and orange [8]. Many studies have shown that the petal pigments in camellias are mainly derived from anthocyanins, with the red color being mainly from anthocyanins of the cyanidin glycosides [9], while the yellow pigment is mainly from the quercetin glycosides [8]. Although there have been some studies on the genes regulating petal flower color in different ornamental camellias [10], little is known about the molecular mechanisms underlying floral color domestication.

In addition to the variations in floral shape and color, the flowering time and period is another important trait in the breeding of camellias. And obtaining hybrids using parents with different flowering periods is the main means of breeding varieties with extended flowering periods. *C. azalea* is a newly discovered species closely related to *C. japonica* with an extraordinary long blooming period (about ten months a year), which has been wildly used as a parent for the breeding of new varieties [11]. Although hybridization of *C. azalea* and *C. japonica* varieties has successfully produced many F_1_ hybrids with extended flowering periods [11], the underlying molecular basis for regulating seasonal flowering remains unknown. Today, more than 40,000 records of cultivars are documented in the Database of International *Camellia* Register [12]. The remarkable diversity of camellias provides a rich resource for understanding the genetic regulation of ornamental trait formation and breeding. However, the study of understanding evolutionary and breeding history has become more complex due to frequent genomic polyploidy and interspecific hybridization events in cultivated camellias [13].

The availability of a high-quality reference genome sequence is important for integrating the large-scale genomic, transcriptomic, and epigenetic research to understand the formation of ornamental traits. Here, we present a chromosome-level genome reference sequence of wild *C. japonica* (cjaND). To validate the reference genome, we further construct a high-density genetic linkage map through re-sequencing the hybrids of *C. japonica* and *C. chekiangoleosa* for future genetic dissection of ornamental traits. Evolutionary analyses reveal that ancient whole-genome duplication and the large-scale segmental duplication of chromosome may contribute to the formation of diverse floral patterns. By comparing the year-round gene expression profiles, we have identified the gene co-expression networks underlying seasonal flushing and flowering and suggest that the regulatory module, including *GIGANTEA* (*GI*), *CONSTANS* (*CO*), and *FLOWERING LOCUS T* (*FT*) genes, may be responsible for mediating the environmental cues to determine seasonality of growth and flowering. This reference sequence is an important resource for understanding the formation of ornamental traits; it will also promote breeding by developing molecular markers associated with traits.

## Results

### Genome assembly and construction of a genetic linkage map for C. japonica

The wild *C. japonica* “Naidong” (cjaND) plant was initially identified for the genome sequencing analysis (Fig. 1a). We showed that cjaND is diploid with 30 chromosomes (2n = 2x = 30, Additional file 1: Fig. S1a), which is consistent to previous analyses [13]. We estimated the cjaND genome to about 2.93 Gb in size with a genome heterozygosity of 1.45%, based on the Kmer analysis of the short-reads sequences (Additional file 1: Fig. S1b; Additional file 2: Table S1). To construct a chromosome-scale reference genome of cjaND, we used a combination of Illumina short-read sequencing and PacBio long-read sequencing technologies. We obtained the initial de novo assembly by using 259.94 Gb PacBio long-reads sequences and further corrected the assembly using the short reads. By high-resolution chromosome conformation capture analysis (Hi-C) (Additional file 2: Table S2), we anchored and sequenced 2.59 Gb of sequences (94.57% of all mapped sequences) to pseudochromosomes (Additional file 1: Fig. S2; Additional file 2: Table S3). Finally, we constructed the reference genome of 2.80 Gb sequences with contigs N50 of 510.94 Kb and Scaffold N50 of 175.51 Mb (Additional file 2: Table S4). We evaluated the basic genomic features of the cjaND genome to gain the overall information of *C. japonica* (Fig. 1b).

**Fig. 2:**
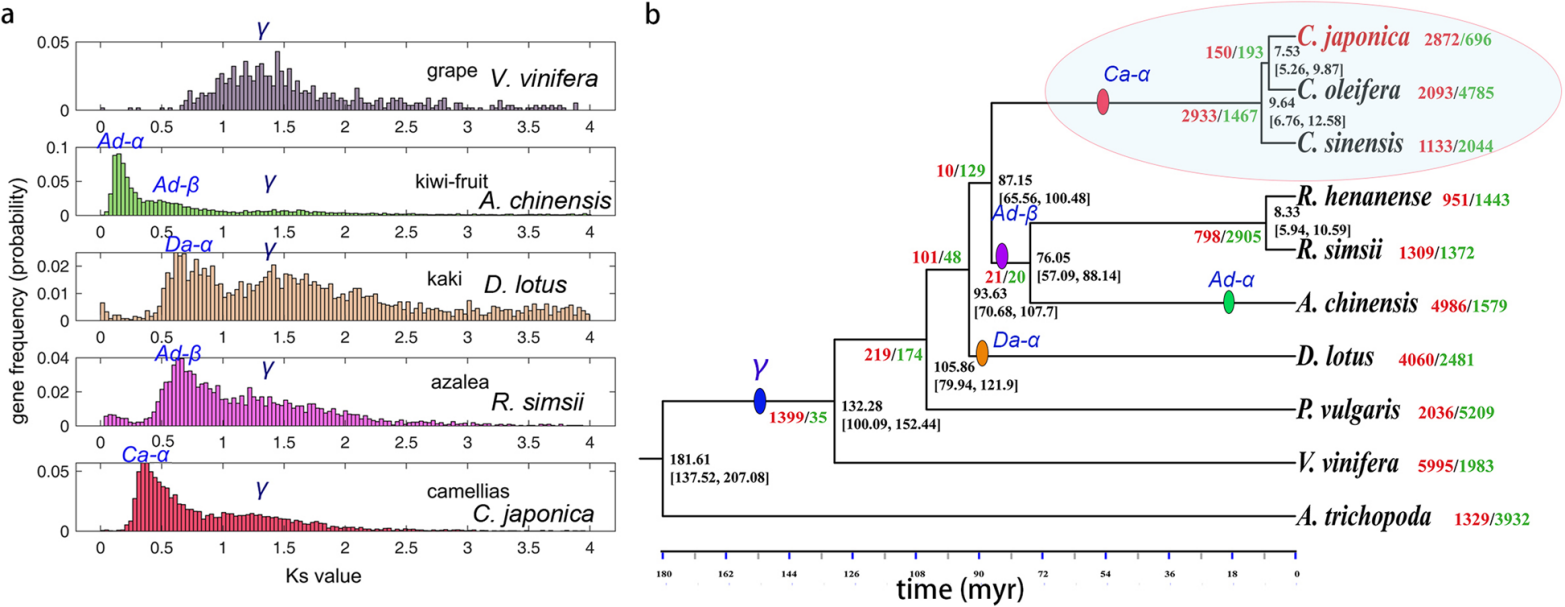
The construction of genetic linkage map in *Camellia*. **a** A genetic linkage map containing 4255 markers is constructed based on F_1_ hybrids from *C. japonica* and *C. chekiangoleosa*. **b** The floral morphology of *C. japonica* (left) and *C. chekiangoleosa* (right). **c**, **d** The violin plot of the floral size (**c**) and stamen numbers (**d**) for the parents and F_1_ offspring (*n* = 10), respectively. The flower size and stamen number of *C. chekiangoleosa* are significantly larger than those of* C. japonica*

Based on the cjaND genome sequences, a cross-population of two closely related *Camellia* species, *C. japonica* and *C. chekiangoleosa*, was examined by the Specific locus amplified fragment sequencing (SLAF-seq) method to construct a genetic linkage map. In total, we sequenced 100 SLAF libraries (including 98 F1 individuals and two parents), which generated approximately 585.81 Gb clean reads (Additional file 2: Table S5). The average depth was 16.12-fold for F_1_ offspring and 77.47-fold for the parents. The cjaND genome was used as a reference for the identification of SNPs; a total of 15,698,860 SNPs were obtained, and of these, 482,643 were found to conform to the specific genetic segregation configurations (Additional file 1: Fig. S3; Additional file 2: Table S6). After filtering and correction, we obtained a high-density genetic linkage map containing 4255 markers (Fig. 2a; Additional file 2: Table S7). The genetic map consisted of 15 linkage groups (LGs) and covered a total of 2481.24 cM with an average inter-locus distance of 0.59 cM (Additional file 2: Table S7). Considering that *C. japonica* and *C. chekiangoleosa* have significant differences in flower size, floral organ number, and other morphologies (Fig. 2 b–d), we believe that this high-density linkage map presents a valuable resource for genetic dissection of ornamental traits in the future.

### Annotation and assessments of the cjaND genome

We predicted and annotated the repetitive sequences of the cjaND genome using multiple tools. We uncovered 2.2 Gb of repeat sequences, accounting for 78.91% of the assembled sequences (Additional file 2: Table S8). We showed that the *Gypsy* retrotransposon is the most dominant transposon accounting for 23.64% of the genome (Fig. 1c; Additional file 2: Table S8), and in class II DNA transposons, the terminal inverted repeat (TIR) is most abundant accounting for 5.99% of the genome (Fig. 1d; Additional file 2: Table S8).

To identify coding sequences, the cjaND genome was analyzed through the ab initio prediction, homology-based prediction, and transcriptome alignment approaches. In total, we obtained 41,890 gene models with an average gene length of 6344 bp (Additional file 2: Table S9, S10). We found that the length of the gene model was positively correlated with the number of exons (Fig. 1e), and the total intron length was over three times more than the exon length (Additional file 2: Table S10). These results suggest that the emergence of large gene models may be associated with the proliferation and expansion of introns in the cjaND genome. We further assessed the completeness of cjaND genome through the BUSCO (Benchmarking Universal Single-Copy Orthologs) analysis. We showed that cjaND genome is highly competent with complete BUSCOs of 90.28% containing 1028 single-copy genes and 272 multiple-copy genes (Additional file 2: Table S11).

### The evolutionary history reveals a lineage-specific WGD in cjaND

**Fig. 3:**
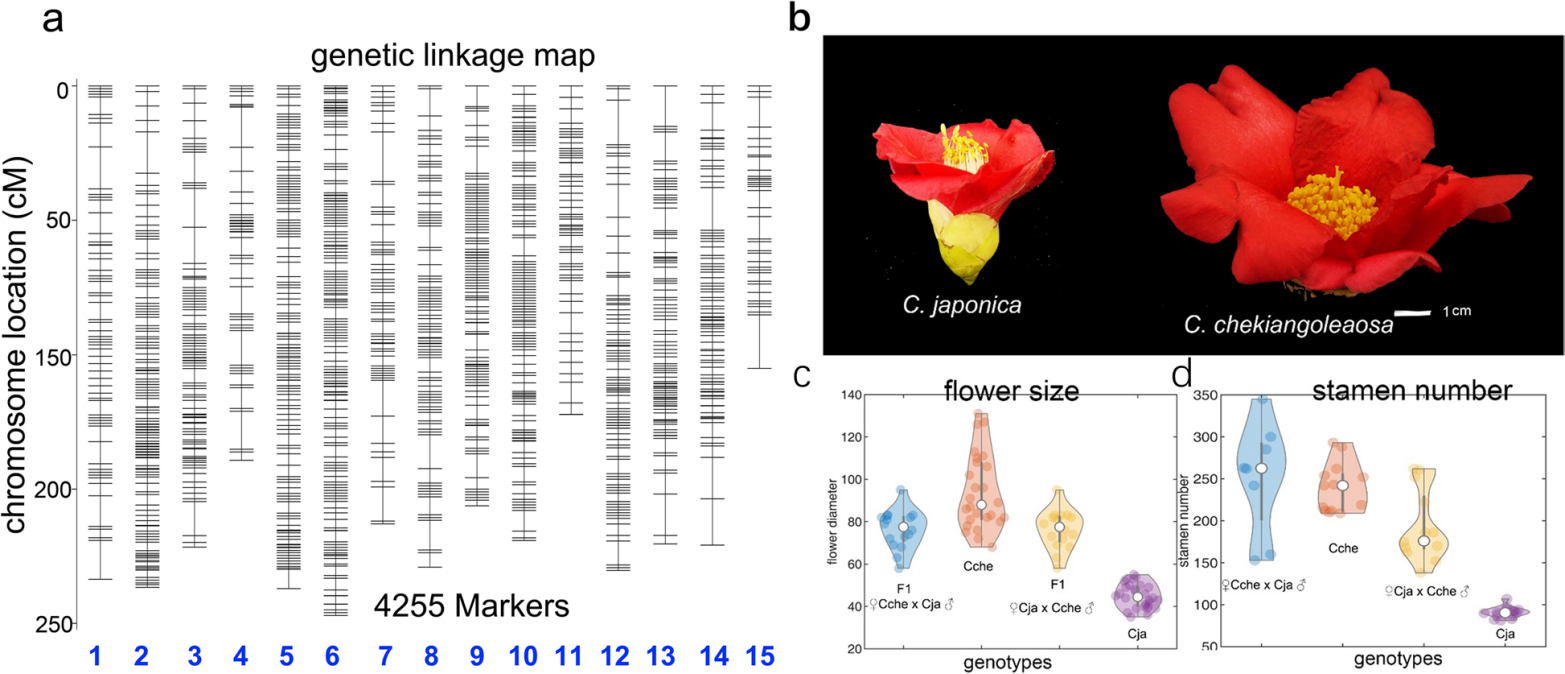
Evolution of the WGD events and phylogenetic analysis of cjaND genome. **a** Distribution of *Ks* of syntenic gene-pairs in camellias (*C. japonica*), azalea (*Rhododendron simsii*), kaki (*Diospyros kaki*), kiwi fruit (*Actinidia chinensis*), and grape (*Vitis vinifera*). γ labels the ancient gamma WGD; Ad-β labels the common WGD for kiwi fruit and azalea; Ad-α labels the recent lineage-specific WGD in kiwi fruit; Da-α labels the lineage-specific WGD in kaki; Ca-α labels the Camellia specific WGD. **b** A time-calibrated phylogenetic tree of species based on the analysis of whole-genome orthologous genes. The red circle indicates the assessed species in *Camellia* genus. Color-filled dots indicate the postulated incidences of WGD events based on the *Ks* distribution patterns. *C. japonica* and *C. oleifera* have diverted about 7.53 million years ago

To gain insights of the origin and evolution of *C. japonica*, we investigated the gene family and phylogenetic relationships using close and distant plant species. Consistent to previous studies in other *Camellia* species [14, 15], we uncovered two whole-genome duplication events (WGDs) in cjaND genome: the gamma triplication and a more recent duplication event (Fig. 3a). Although this recent WGD event was previously predicted to be a shared event among *Actinidia* and *Camellia* [16], we showed that it (named as Ca-α) is likely *Camellia*-specific based on the occurrences of synonymous mutation rate (Ks) in close-related Ericales (Fig. 3a). We found that there was a subtle shift of Ca-α *Ks* peak compared to azalea (*Rhododendron simsii*), kaki (*Diospyros kaki*), and kiwi fruit (*Actinidia chinensis*) (Fig. 3a). To further evaluate the WGDs, we constructed a phylogenetic tree using single-copy orthologs; our result is consistent with the taxonomic placement of all assessed plant species (Additional file 2: Table S12; Fig. 3b). We positioned the WGDs according to the phylogenetic relationships of species and *Ks* distributions and showed that Ca-α is more recent than the Ad-β event that is shared among *Rhododendron* and *Actinidia* (Fig. 3b). Furthermore, our time-calibrated results indicated that *C. japonica* and *C. oleifera* are evolutionary closer and have diverged around 7.53 million year ago (Fig. 3b).

### Duplication of chr.8 gives rise to a duplicated AG locus in cjaND

**Fig. 4:**
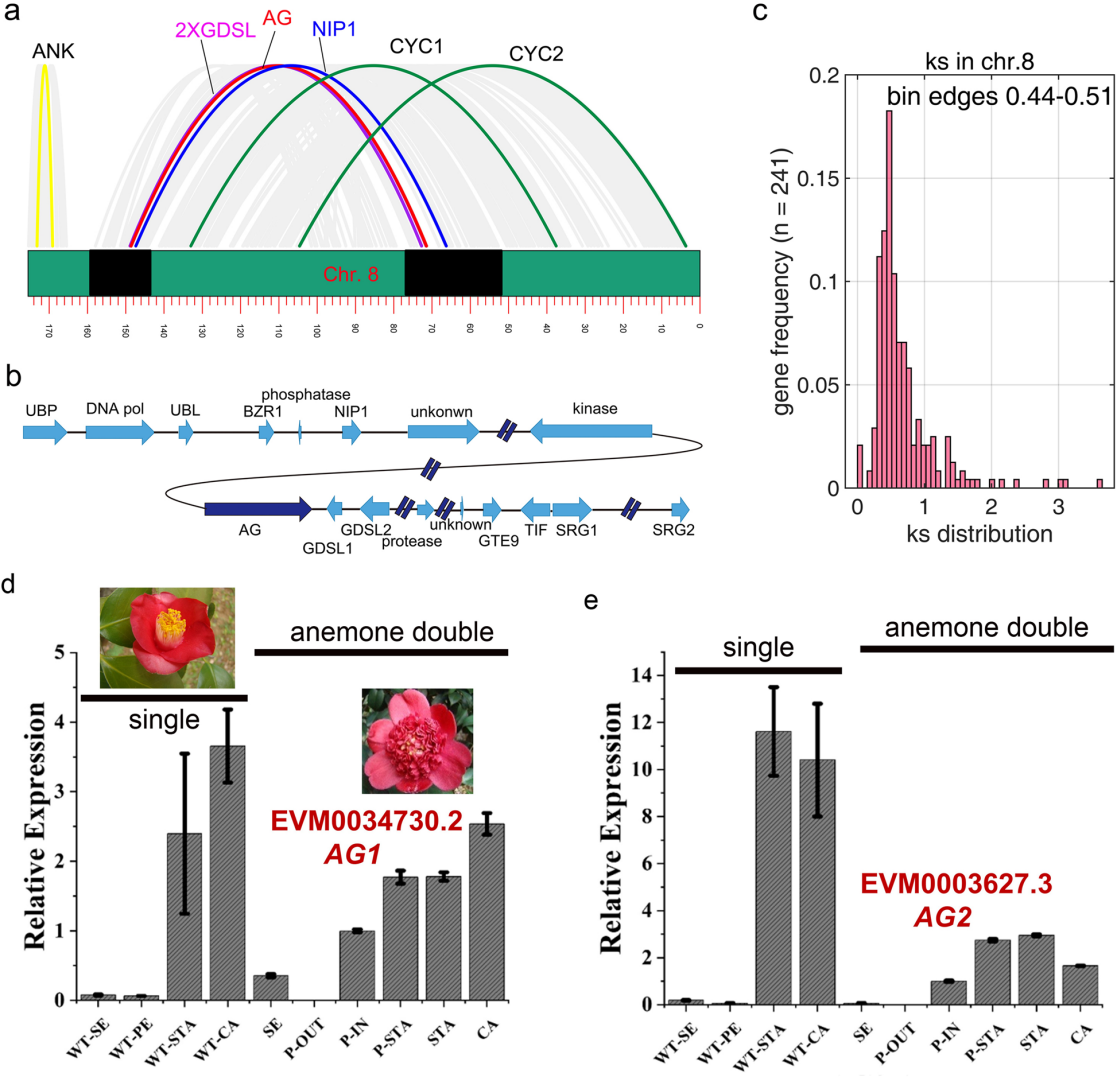
The micro-collinearity analysis reveals the history of segmental duplication of chr. 8, which gives rise to a duplicated *AG* locus in cjaND. **a** The syntenic analysis of chr. 8 based orthologous gene-pairs. The gray and colored curves indicate the syntenic pairs of genes. The gene labels on the top are conserved genes associated with *AG* in ancestors of eudicots. The rectangle on the bottom indicates the chr.8 and the black shading areas indicate the syntenic blocks containing the two *AG* paralogs, within which the conserved two *GDSL* genes and *NIP1* genes are found. **b** The representative genomic structure of the syntenic block containing *AG* genes, and only genes with orthologous pairs are shown. The length of arrow shapes is on scale with the length of gene model. The scale of intergenic region is ten times that of the gene coding region; the interrupted line indicates the length is longer than 1 Mb, and the details of gene locations are in the Supplementary Table S13. **c** The distribution of *Ks* values of syntenic gene-pairs in chr.8, and the top bin of *Ks* values is between 0.44 and 0.51. **d**–**e** Relative expression level of *AG* copies of *C. japonica*. *CjAG1* (**d**) and *CjAG2* (**e**) display similar expression patterns in single (cjaND) and doubled flowers (*C. japonica* “JINPANLIZHI”). SE, sepal; PE, petal; STA, stamen; CA, carpel; P-OUT, outer petal; P-IN, inner petal

We found that the chr.8 has a large area of collinearity within itself (Fig. 1b). To further investigate the micro-collinearity, we characterized the syntenic blocks within chr.8 and uncovered 11 blocks containing 481 orthologous genes (Fig. 4a), with the regions of the syntenic blocks covering 89.88% of the whole chromosome. We found that one of the duplication segment contained the C class homolog (*CjAG*), a key regulator involved in the formation of double flowers [7], resulting in two copies of the C-function genes (Additional file 2: Table S13; Fig. 4a, b). Phylogenetic and sequence analyses revealed that *CjAG1* and *CjAG2* are paralogs derived from the duplication events of AG lineage in genus *Camellia*, including *C. japonica* and *C. sinensis* (Fig. S4). Comparative studies have shown that the C-function genes retain a high degree of collinearity in higher plants in terms of gene content and gene order [17]. We showed that, in chr.8 of cjaND genome, the duplicated *AG* locus shared some conserved orthologs (landmark genes) [17] with the eudicot ancestor (Fig. 4c), including *NIP1*, *GSDL*, *ANK*, and *CYC*, but some related genes (*ANK* and two *CYC*) are separated to different syntenic blocks in *C. japonica* (see Additional file 2: Table S13 for details of gene information; Fig. 4b). We further evaluated the *Ks* distribution of paired paralogs in chr.8 and showed that the *Ks* peak is coincided with the Ca-α peak, suggesting duplication of chr.8 is coinciding with the Ca-α WGD. These results indicated that chr.8 has undergone a large duplication event which generates two C-function genes. To understand the functional significance of duplication, we investigated the expression pattern in single and doubled flowers and found that the two *AG* copies had similar expression in inner floral organs of stamen, carpel, and inner petals of anemone double flower (Fig. 4d, e), which suggests both copies are functional in *C. japonica*. We further analyzed the co-expression network of the two *AG* copies by using the previous floral transcriptome dataset containing 11 floral tissue types from single, formal double, and anemone double flowers [6]. We found that *CjAG1* and *CjAG2* had formed different regulatory networks involved in floral development (Additional file 1: Fig. S5). These results together indicate that the duplication of *AG* paralogs may be one of the key points for domestication of floral shapes.

### Annual gene expression profiling in C. japonica and C. azalea provides the molecular basis of seasonality

**Fig. 5:**
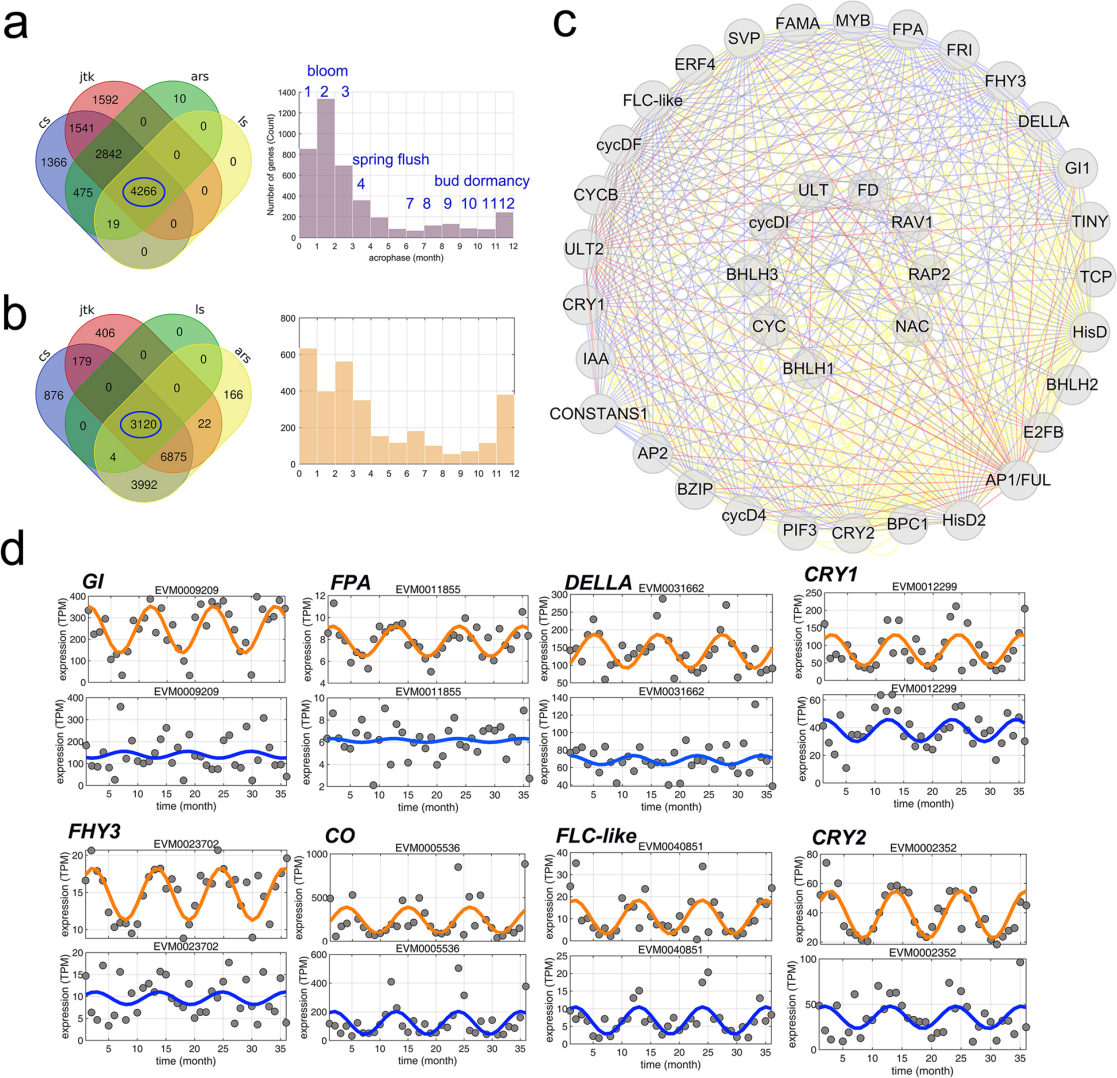
The comparative analysis of year-round transcriptomics in *C. japonica* and *C. azalea* reveals the genes with annual rhythms and their co-expression networks. The identification of rhythmic genes based on four different models based on the year-round gene expression data in *C. japonica* (**a**) and *C. azalea* (**b**) respectively, and genes with significant correlations (*P*-value < 0.05) are recovered for each model and common set of genes are identified as the confident candidates of rhythmic genes. The right panel indicates the distribution of acrophase for the common rhythmic genes. In *C. japonica*, the identification of rhythmic genes in different months is potentially associated with key events including blooming in January to March, spring flush in April and floral bud dormancy from July to December. **c** The sub-network is identified from the co-expression modules by the WGCNA analysis using all rhythmic genes, in which the genes with important regulatory functions are uncovered from the common set of genes of *C. japonica* and *C. azalea* (Table S14) and selected based on connectivity and correlations. The line colors indicate biological pathways related to the candidate genes. Red, floral development; blue, flowering time; yellow, light signaling; gray, other transcription factors. **d** The identification of genes with eliminated or disrupted annual rhythms between *C. japonica* and *C. azalea*. In each panel, the expression levels (TPM) of each candidate gene are scattered as gray dots, and orange (*C. japonica*) and blue (*C. azalea*) lines indicate the fitted rhythmic models

Wild *C. japonica* usually blooms in late winter and early spring each year, while *C. azalea* displays a long flowering period, lasting up to 10 months. To study the molecular basis of seasonal flowering, we conducted annual transcriptomics analyses and obtained the monthly gene expression profiles for *C. japonica* and *C. azalea* throughout a whole year. We used four different methods to obtain the seasonal expressed genes for *C. japonica* and *C. azalea* respectively (Additional file 1: Fig. S6, 7), and the distribution of acrophase of rhythmic genes may be associated with seasonal activities, such as blooming, flushing, and other growth events (Fig. 5a, b). We hypothesize that some essential pathways that convey the seasonal signals (e.g., day-length and temperature) may play a decisive role in controlling growth or flowering.

Based on comparative analysis, we found that majority of the rhythmic genes are altered in *C. japonica* and *C. azalea*, and 730 genes maintained the rhythmic expression (Additional file 1: Fig. S8a, b). To further investigate the genome-wide gene expression profiles, we performed the weighted gene co-expression network analysis (WGCNA) [18] in *C. japonica* using genes that display seasonal rhythmicity in *C. japonica* or *C. azalea* (Fig. 5a, b). We uncovered 11 distinct co-expression modules that may be involved in specific seasonal activities (Additional file 1: Fig. S8c; Fig. S9). We focused on the genes displaying seasonal rhythms in both *C. japonica* and *C. azalea* and showed that pathways involved in light signaling, flowering time, seasonal growth, and cell cycle form a complex and intertwined sub-network that contained a number of key regulatory genes including *GI*, *CONSTANS*, *APELATA1*, *TCP*, and others (Additional file 2: Table S14; Fig. 5c). Through comparing the expression profiles, we found that the homologs of *GI*, *FPA*, and *DELLA* did not maintain the periodic expression patterns in *C. azalea*, and homologs involved in the far-red light signaling, *CRYPTOCHROME 1* (*CRY1*) and *CRYPTOCHROME 2* (*CYR2*), and flowering time, *FAR-RED ELONGATED HYPOCOTYLS 3* (*FHY3*), *CO*, and *FLOWERING LOCUS C* (*FLC*) have shown weakened and shifted rhythmicity (Fig. 5d).

**Fig. 6:**
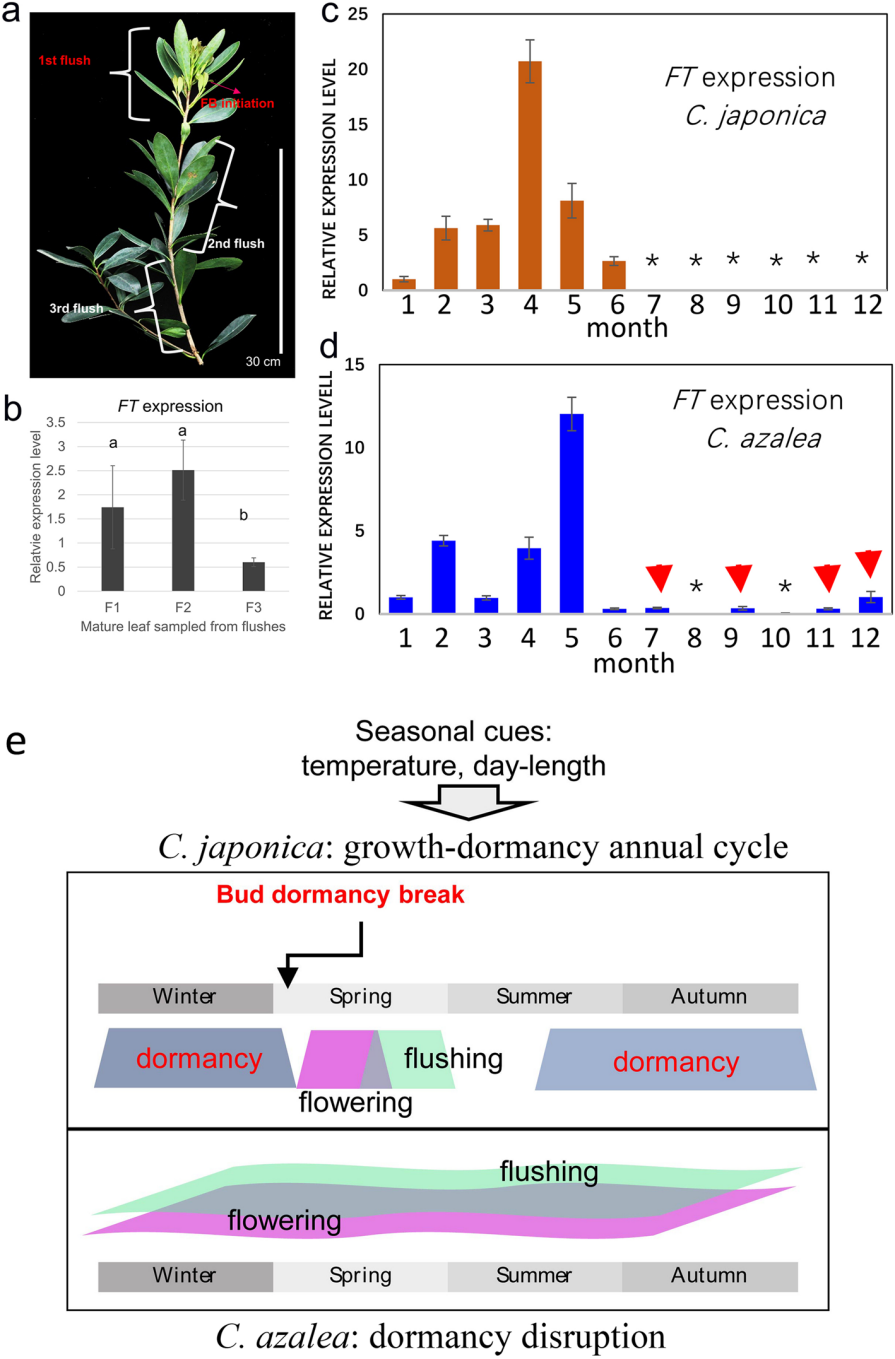
Seasonal expression of the *FT* ortholog and a working model of seasonality in *C. japonica* and *C. azalea*. **a** A representative photo of a *C. azalea* at flushing and floral bud initiation. Due to the continuous flushing and flowering, the periodic flowering activities can be traced based on the location of flush. **b** The expression of *FT* in mature leaves of different batches of flushes. The mature leaves from second flush are obtained year-round to detect the expression of *FT* in *C. japonica* (**c**) and *C. azalea* (**d**). One-way ANOVA and multiple comparisons are performed for significance tests (*p*-value < 0.01), and different letters indicate significant changes. The stars indicate not detectable of expression. Subtle and dispersed expression of *FT* during summer and autumn is indicated by red arrowheads in *C. azalea*. **e** Combining the previous results together, we propose the seasonal cues (e.g., daylength, temperature, etc.) can be perceived and conveyed to regulate annual vegetative and reproductive growth in *C. japonica*, while in *C. azalea*, the flushing and flowering processes are continuous and fluctuating mainly due to the missing or disruption of the bud dormancy. The growth processes are mainly manifested as flowering (red), bud dormancy (dark blue) and flushing (green), which are color shaded

The *FLOWERING LOCUS T* (*FT*) genes in woody perennial poplars have been shown to play a central role in regulating seasonality of vegetative and reproductive growth [19, 20]. To obtain the *FT* orthologs in *C. japonica* and *C. azalea*, we cloned the full-length coding sequences based on transcriptome sequences (Additional file 1: Fig. S10a; Additional file 2: Table S15). Phylogenetic analysis indicated *CjFT* and *CaFT* were orthologs of *FT*, despite that there were minor changes in their coding sequences (Additional file 1: Fig. S10a, b). Considering the continuous flowering of *C. azalea*, we characterized the expression levels of the *FT* orthologs in mature leaves from different flushes at the onset of floral bud initiation (Fig. 6a). We revealed that *FT* expression is detected in all flushes but mainly in the first and second flushes (Fig. 6b). We further investigated the annual expression of *FT* ortholog in *C. japonica* and *C. azalea*. We showed that the *FT* expression in *C. japonica* maintained from January to July, peaked in April, and disappeared from July to December (Fig. 6c), whereas in *C. azalea*, *FT* was detected at high levels in May and expressed dispersed throughout the year (Fig. 6d). These results suggest that the *FT* gene may play a regulatory role in the periodic flowering and vegetative growth of camellias. We propose that the seasonal signals such as photoperiod signals and temperature are involved in regulating seasonal growth and development through establishing annual cycle of bud dormancy and break (Fig. 6e).

## Discussion

Our chromosome-level genome reference for *C. japonica* is of high quality and allows comparative genomics analyses to reveal the evolution and domestication of camellias. We identified two WGD events in cjaND genome, which are consistent with other studies of *Camellia* species [21], and our results show that the most recent WGD event is not a shared event for Ericales, but a lineage-specific one in related species of genus *Camellia* (Fig. 3). This concept fits well with previous studies of closely related species azaleas and tea, suggesting that *Camellia* species undergoes a unique WGD [22]. Further research on species of Theaceae, such as *Schima*, could shed more detail into the origin and timing of this WGD event.

Gene duplication is an important source for the breeding of diverse ornamental traits [23]. For instance, the recently duplicated paralogs of *FT* have been shown to be involved in regulation of flower timer divergence in domesticated sunflowers [24, 25]. Our micro-collinearity analysis reveals that chr. 8 arose from a large segmental duplication that probably occurred simultaneously with the recent WGD event in *C. japonica* (Fig. 4). We found that regional duplication produced at least 240 homolog pairs (Fig. 4c), and this result provides a basis for studying gene functional differentiation and mutation accumulation in paralogs. We speculate that functional differentiation of homologs (e.g., subfunctionalization or neofunctionalization) may underlie selection for the remarkable ornamental traits in varieties of *C. japonica*. We found that the segmental duplication yielded two functional *AG* loci that may be involved in the formation of diverse floral forms in camellias. How do these two *AG* paralogs contribute to the domestication of different double-flowers? We showed that the expression patterns of *AG* paralogs are similar in wild cjaND and anemone double flowers, indicating redundant roles in the regulation of floral development [6, 7]. Previous studies in *C. japonica* have shown that an *AG* copy (*CjAG*) has a conserved C function in regulating floral development and is associated with double flower formation, but its expression pattern is not consistent in different types of double flowers [7]. We showed that the expression patterns of *CjAG1* and *CjAG2* had subtle differences (Fig. 4e) and may result in different regulatory networks involved in floral development (Additional file 1: Fig. S5). These results in part suggest that potential degenerative mutations of paralogs may be linked to the morphological changes [26]. Studies targeting functional differences and patterns of sequence variation in paralogs may provide informative insights on the future breeding processes. Therefore, we postulate that these two copies of *AG* genes may have subtle functional diversification in regulating the development of inner floral organs. A detailed analysis of the two duplicated loci and expression profiling is needed to sort out the contribution of the formation of double flower forms.

Our comparative analysis of annual gene expression provides molecular insights into the seasonality of vegetative and reproductive growth. *C. japonica* is a tree species with typical annual rhythms of growth: it fully blooms in early spring and starts flushing in April, and its flower buds are developed during summer and enter to a period of about 6-month dormancy until flowering the next year [11, 27]. *C. azalea* blooms several times a year, mainly due to the continuous formation of floral buds after flushing, and flower buds can directly enter flowering processes without dormancy [27]. By comparing the genome-wide gene expression patterns of *C. japonica* and *C. azalea*, we revealed that the expression patterns of a large number of rhythmic genes changed in these two species (Fig. 5a, b), indicating that global gene expression is closely related to the seasonal growth and development. Combined with gene co-expression network and functional analyses, we identified a co-expression sub-network containing key regulators involved in the pathways of photoperiod signaling, plant hormone, and flowering time (Fig. 5c). We showed that some genes involved in the regulation of cell cycle and histone modification (Additional file 1: Fig. S9) can maintain rhythmic expression, and some regulatory genes of signaling display altered or abolished rhythms in *C. azalea* (Fig. 5d). These results indicate the alteration of genes involved in the signal transduction processes may play an important role of continuous flowering in *C. azalea*.

As a perennial woody flower, continuous flowering is an ornamental trait of great significance. The *FT* gene has been extensively identified as a central regulator of seasonal growth and flowering in perennials [19, 20]. Our expression analysis of *FT* orthologs suggests that expression changes in *C. japonica* and *C. azalea* may be critical for determining the activity of flowering signals (Fig. 6), and as potential upstream factors of *FT*, *CO* and *GI* may play a role in mediating environmental factors [28]. Correspondingly, we found that *GI* expression levels lost periodically in *C. azalea* and maintained consistently high expression levels (Fig. 5d). Taken these results, we provide a model (Fig. 6e) in which the seasonal cues, including photoperiod signaling or temperature, are involved in the regulation of annual growth activities (e.g., flushing and flowering) in *C. japonica*, while in *C. azalea*, the continuous flushing and flowering processes are mainly related to the disruption of the formation and maintenance of bud dormancy (Fig. 6e). Although the trait of continuous flowering has been elaborated in some flowers (e.g., *Rosa* [29, 30], *Prunus* [31], etc.), the signaling processes that regulate flowering and flower bud dormancy are poorly understood. In the future, comparative genomics studies may be one of the solutions to discover the factors underlying the process of continuous flowering in *Camellia* species.

*C. japonica* is a unique ornamental flower in the genus *Camellia* and an important member for comparative genomics analysis of ornamental trait formation and domestication. Recently, the genomic information on several *Camellia* species with different traits has been available, including *C. chekiangoleosa* [32] and *C. lanceoleosa* [33], which provides a solid basis for understanding the molecular basis of trait formation. Cultivated camellias has a variety of ornamental traits, including leaf morphology, flower color, flower morphology, floral scent, and flowering time. The current genomic reference of cjaND, together with a high-density of genetic linkage markers, provides a genomic platform for integrating genomic resources such as gene expression, DNA modification, and genome structure of various varieties of camellias around the world.

## Conclusions

*Camellia japonica* is a famous ornamental flower with a long history of cultivation and multifarious floral shapes and colors. Here, we provided a high-quality reference genome and a high-density genetic linkage map through combing multiple genome sequencing analyses. We show that whole-genome duplication and segmental duplication play pivotal roles in the formation of diverse floral patterns. In addition, we demonstrate that modification of annual expression profiles of key regulators (e.g., *GI*, *CO*, *FT* and etc.) is fundamental for achieving seasonality.

## Methods

### Plant materials

Plants of *Camellia japonica* L. (cjaND) and *C. azalea* were grown at the Research Institute of Subtropical Forestry under natural conditions (119° 57′ N, 30° 04′ E; Fuyang city, Zhejiang, China), and leaves are used for DNA and RNA sample preparations.

For the construction of genetic linkage map, ninety-eight F_1_ individuals of the cross-population of cjaND and *C. chekiangoleosa* were obtained and preserved in RISF, and the tender leaves of all individuals were harvested for DNA sample preparation.

For annual gene expression, leaves from 2nd flush of cjaND and *C. azalea* at each month were collected and frozen immediately in liquid nitrogen and then kept at – 80 °C before use. From March 2017 to February 2018, we harvested leaves and stored them for RNA sequencing. Samples were collected from 9:00 a.m. to 10:30 a.m. on the 15th of each month.

### Genome sequencing and assembly of Camellia japonica genome

#### Genome survey

The genomic DNA of the cjaND plant was extracted according to a CTAB method [34] by using the young leaves. We constructed eight 270 bp Illumina libraries using the genomic DNA of *Camellia japonica* according to the manufacturer's protocol, and each library was sequenced using a 150-bp paired-end sequencing strategy on the Illumina HiSeq X ten platform (Illumina, CA, USA). Then, we used Trimmomatic [35] (v0.32) software to remove the adapters, reads containing more than 3% ambiguous bases (N), reads containing more than 50% *Q* < 30, and other low-quality sequences of the raw data. Finally, we used the clean data to estimate the genome size, heterozygosity, and the ratio of repeats by the Jellyfish [36] (v1.1.12) program with the parameters kmer = 21.

#### PacBio sequencing and de novo assembly

The construction of the PacBio library was following the user’s manual. BluePippin was used for target fragment screening. Qubit2.0 (Thermo Fisher Scientific, MA, USA) and Agilent 2100 (Agilent Technologies, CA, USA) were respectively used to detect the concentration and Insert Size of all the libraries, and the Q-PCR method was used to accurately quantify the effective concentration of the libraries. The high-quality libraries were sequenced using the PacBio platform (Pacific Biosciences, CA, USA). The clean reads were firstly corrected using Canu software [37], and then the corrected data were assembled using Canu [37] (v1.8), Falcon [38] (v0.3.0), and Wtdbg software with default parameters, respectively. The Wtdbg assembled genome was optimized with Canu and Falcon assembly results by Quickmerge software [39], and the optimized genome was aligned using Numer software to remove redundant sequences. Finally, Pilon [40] software was used to correct errors in combination with Illumina short reads.

#### Hi-C sequencing and genome assembly

Previous analysis in Camellia tissues have shown that tissue culture materials were suitable for chromatin isolation [26, 41]. To obtain tissue samples and reduce potential contaminations for Hi-C analysis, we generated sterile shoots by the tissue culture method. Briefly, the young leaves of cjaND were sterilized with bleach (10% NaClO, 0.025% Triton X-100) for 15 min and 70% ethanol for 45 s. Then, Murashige-Skoog medium containing 2,4-dichlorophenoxyacetic acid (2,4-D) at 0.5 mg l^−1^ and coconut water at 50 ml l^−1^ were used for callus induction. The MS medium containing thidiazuron (TDZ) at 1 mg l^−1^, 1-naphthylacetic acid (NAA) at 0.3 mg l^−1^, phenylacetic acid (PAA) at 15 mg l^−1^, and coconut water at 50 ml l^−1^ were then used for re-differentiation of calli. And the young shoots were further cultured on MS medium with NAA 0.5 mg l^−1^, 6-benzylaminopurine 1.0 mg l^−1^, and coconut water 50 ml l^−1^. Formaldehyde was used for cross-linking, and interacting DNA fragments were cycled; then, the DNA was de-cross-linked, purified, and fragmented into 300 bp–700 bp. After Hi-C libraries passed quality control, the Illumina Hiseq platform (Illumina, CA, USA) was used for paired-end 150 bp sequencing. Raw data were filtered to remove adaptors and low-quality reads.

LACHESIS [42] (https://github.com/shendurelab/LACHESIS) software was used for chromosome-scale assembly with the parameters as “CLUSTER MIN RE SITES = 22, CLUSTER MAX LINK DENSITY = 2, CLUSTER NONINFORMATIVE RATIO = 2, ORDER MIN N RES IN TRUNK = 10, ORDER MIN N RES IN SHREDS = 10”. Pbjelly [43] was used to fill the gap. To investigate the accuracy and integrity of the chromosome-scale assembly, firstly, Hi-C data were evaluated by HiC-Pro software [44].

### Construction of a genetic linkage map by SLAF (specific locus amplified fragment) sequencing

#### SLAF-seq

Taking the final assembled genome of *C. japonica* as a reference, the most suitable enzyme digestion scheme for the genome of *C. japonica* was selected [45, 46]. After the libraries were qualified, paired-end 125 bp sequencing was performed on Illumina HiSeq X ten (Illumina, CA, USA). The reads with a length of 1–126 bp were considered valid data. To ensure the quality of reads, after trimming the adapters, the reads with ambiguous bases (N) of more than 10% were removed.

#### SNPs identification and genotyping

GATK [47] (The Genome Analysis Toolkit) software was first used for InDel realignment, that is, the loci near the insertion and deletion alignment were locally re-aligned to correct the error caused by insertion and deletion. Then, GATK and samtools were used for mutation detection, mainly including SNP and InDel. According to the parents of each SNP, the parents were first genotyped, and then the genotypes of the offspring were determined according to the consistency of the sequence of the offspring with the parents. These segregation patterns, except aa × bb, were used as valid markers.

#### Construction of genetic linkage map

High-quality SNP markers were screened for subsequent map construction and analysis. The criteria for marker filtering were as follows: (1) offspring sequencing integrity of more than 60% (for a single polymorphic marker locus, less than 60 individuals out of 100 offspring have a definite genotype); (2) segregation distortion marker (*p* < 0.0001, chi-square test); (3) parental sequencing depth more than 10X. Genetic map construction was performed by HighMap software. First, the recombination rate and MLOD value between markers were calculated using Kosambi’s mapping function, and the order of the initial version of the markers was obtained by the maximum likelihood method, and then the correction of the genotyping was performed, including mapping, correction, mapping again, and correction again, and finally a high-quality genetic map was obtained.

### Genome annotation

Based on LTR FINDER [48] (v1.05, https://github.com/xzhub/LTR_Finder), MITE-Hunter [47], RepeatScout [49] (v1.0.5), and PILER-DF [50] (v2.4) software, we constructed the genome repeat sequence database according to the principle of structure prediction and de novo prediction. The PASTEClassifier [51] software was used to classify the database and then merged with the Repbase database [52] as the final repeat sequence database. Then, RepeatMasker [53] (v4.0.6) was used to predict the repeat sequence based on the constructed repeat sequence database.

Three different strategies were used for the prediction of gene structure, mainly ab initio prediction, homologous species-based prediction, and Unigene based prediction, and the prediction results were finally integrated using the EVM [54] (v1.1.1) software. Ab initio prediction was performed using Genscan [55], Augustus [56] (v2.4), GlimmerHMM [57] (v3.0.4), GeneID [58] (v1.4), and SNAP [59]. Homologous species-based prediction using GeMoMa [60] (v1.3.1). Hisat [61] (v2.0.4) and Stringtie [62] (v1.2.3) were used for assembly with reference transcripts, and TransDecoder [63] (v2.0) and GeneMarkS-T [64] (v5.1) were used for gene prediction; PASA [65] (v2.0.2) was used for prediction of Unigene sequences based on transcriptome data without reference assembly. Finally, EVM was used to integrate the prediction results obtained by the above three methods.

Non-coding RNAs are non-protein coding RNAs, including microRNA, rRNA, tRNA, and other RNAs with known functions. According to the structural characteristics of different non-coding RNAs, different strategies are adopted to predict different non-coding RNAs. rRNA and microRNA prediction were performed based on the Rfam database [66] and miRBase database [67] respectively, using the Infernal 1.1 [68] (v1.1.2) software. And tRNAscan-SE [69] (v1.3.1) was used to identify tRNAs. In total, 476 rRNAs, 621 tRNAs, and 116 miRNAs were annotated in the whole genome.

### Evaluation of cjaND genome

To investigate the integrity of genome assembly, we used BUSCO [70] (Benchmarking Universal Single-Copy Orthologs, v2.0) software to assess the integrity of *C. japonica* genome assembly. Among the assembled genes, a total of 1300 complete BUSCO genes were found, including 1028 single copies and 272 multiple copies. The BWA-MEM [71] software (v0.7.10-r789) was used to map the short sequences obtained by the Illumina HiSeq X ten sequencing platform to the reference genome, and the mapped rate was 99.49%. The sequence in which both paired-end reads mapped to the reference genome and consistent with the length distribution of the sequencing fragments accounts for 94.37%.

### Evolutionary and phylogenetic analyses

We used Orthofinder v2.5.4 [72] to identify orthogroups of *Actinidia chinensis* [73], *Camellia sinensis*-Tieguanyin [74], *Camellia oleifera* [14], *Rhododendron henanense* [75], *Rhododendron simsii* [16], *Amborella trichopoda* [76], *Vitis vinifera* [77], *Primula vulgaris* [78], *Diospyros lotus* [79], and *Camellia japonica*. A total of 30,433 orthogroups were discovered, containing 77 single-copy orthogroups; *C. japonica* genome contains 39,930 genes in 17,175 orthogroups (Table S12).

The maximum likelihood phylogenetic tree is constructed by 1381 orthogroups with a minimum of 70.0% of species having single-copy genes in any orthogroup using Orthofinder v2.5.4 [72] using the following parameters: –M msa –T raxml. We then conducted phylogenomic dating in the MCMCtree program from PAML v4.9 [80], using *Amborella trichopoda* as an outgroup. The MCMCtree analysis was constructed using 77 single-copy orthogroups, using the following parameters: burn-in-2000, sample-frequency-10, sample number-20,000. For the divergence time estimation, we calibrated the model using divergence time between *Amborella trichopoda* and *Vitis vinifera* (179.0–199.1 Mya), *Actinidia chinensis* and *Rhododendron simsii* (46.6–102.2 Mya), and *Diospyros lotus* and *Camellia sinensis* (81.7–104.9 Mya), obtained from the TimeTree database (http://timetree.org/) [81].

To reveal the expansion and contraction of gene families, we used Cafe5 [82] to count gene numbers at the node in the species tree and infer gene families that had undergone expansions or contractions. We first removed the large gene families with more than 100 gene copies in one or more species. We identified gene family expansions or contractions only when the gene count change was significant with a *P*-value < 0.05.

For the analysis of *Ks* and WGD, we used the MCScanX [83] to perform the syntenic analysis, to obtain homologous gene blocks and gene pairs between and within species. *Ks* estimates for all pairwise comparisons within a gene family were obtained using Yn00 tools from the PAML package [80].

### RNA sequencing for annual gene expression

For expression analysis, we used HISAT2 v2.1.0 [84] to map clean RNA-seq reads to the *C. japonica* reference genome. On average, 81% of the reads map on the genome in *C. azalea* and 85.51% in *C. japonica*. The expression level for *C. japonica* and *C. azalea* (TPM, and expression count data) was obtained using StringTie v1.3.6 [84]. The annual rhythmic genes were identified using the R package DiscoRhythm [85] (v1.2.1). The three biological replicates of annual gene expression are concatenated to form three periods for the identification process.

### Gene co-expression network analysis

The WGCNA R package was used to analyze the co-expression of genes. Co-expression gene modules were identified based on the correlations to the samples; we focused on the annual rhythmic genes that were identified in both *C. japonica* and *C. azalea* (Fig. S7). To further investigate the co-expression module, we combined gene annotation and correlations (cutoff value 0.26) to filter the original networks to obtain the key subnetwork. Next, the Cytoscape software (v3.8.2) was used to visualize the co-expression regulatory network.

### Real-time PCR analysis

Total RNA of cjaND and *C. azalea* was extracted from the leaves using RNAprep Pure Plant Plus Kit (Code No. DP441, TIANGEN, Beijing, China). The first strand of cDNA was synthesized using the TaKaRa PrimeScript™ RT Master Mix Kit (Code No. RR036A, TaKaRa, Dalian, China) according to the manufacturer’s manual. For expression analysis of *FT* genes, we cloned and verified the cDNA sequences of *CjFT* and *CaFT*. The gene-specific primers (Table S15) used in this work were designed by Primer Express 3.0.1 (Applied Biosystems, Foster City, CA, USA). We tested the specificity of qPCR primers by using positive and negative controls and obtained the specific primers for *FT* expression analysis. The RT-qPCR was performed using TB Green® Premix Ex Taq™ II Kit (Code No. RR820A, TaKaRa, Dalian, China) on an ABI PRISM 7300 Real-Time PCR System (Foster City, CA, USA) with three biological replicates. Finally, the gene expression data were analyzed using the 2^−∆∆CT^ method [86].

### Supplementary Information

**Additional file 1: Fig. S1.** The karyotyping and Kmer-based analyses of the cjaND genome. **Fig. S2.** The Hi-C heatmap shows the interaction of the chromosome. **Fig. S3.** The segregation patterns of the genetic makers. **Fig. S4.** The evolution and expression pattern of *CjAGs*. **Fig. S5.** Co-expression network analysis of *CjAG1* and *CjAG2*. **Fig. S6.** Identification of annual rhythmic genes in *C. japonica.* **Fig. S7.** Identification of annual rhythmic genes in *C. azalea.* **Fig. S8.** The relationship of co-expression module of common rhythmic genes. **Fig. S9.** The seasonal expression genes participating in different pathways in *C. japonica* and *C. azalea*. **Fig. S10.** Identification of *FT* genes from *C. japonica* and *C. azalea*.

**Additional file 2: Table S1.** Statistics of Illumina sequencing data of *C. japonica*. **Table S2.** Statistics of Hi-C sequencing data. **Table S3.** Summary of chromosome level assembly based on Hi-C analysis. **Table S4.** Statistics of chromosome level assembly of cjaND genome. **Table S5.** Statistics of SLAF-seq data. **Table S6.** Summary of high-quality SNPs for map construction in each segregation pattern. **Table S7.** Summary and statistics of linkage groups for cjaND genome. **Table S8.** Summary of the identification of repetitive sequences. **Table S9.** Summary of coding gene prediction. **Table S10.** Statistics of gene information of annotated cjaND genome. **Table S11.** Summary of BUSCO evaluation results. **Table S12.** Statistics of the Ortho-group sizes for gene family analysis. **Table S13.** The gene information of syntenic block containing the AG loci in Chr. 8. **Table S14.** The genes of subnetwork based on connectivity and gene annotation. **Table S15.** Primers used in this study

## Data Availability

All supplementary data associated with the paper are available from the online web link. The original sequencing data are deposited in NCBI Bioproject under accession No. PRJNA901631 [87], including genome sequencing data by Illumina (SAMN31724198-SAMN31724205) and PacBio (SAMN31724206-SAMN31724409), Hi-C sequencing (SAMN31724410- SAMN31724412), transcriptome sequencing for genome annotation (SAMN31806788), and annual transcriptome sequencing (SAMN31778641-SAMN31778721). The genome assembly, genome annotation, gene expression data, and source code are also available from Zenodo https://zenodo.org/records/7340615, https://doi.org/10.5281/zenodo.7340615(2022) [88].

## Abbreviations

BUSCO: Benchmarking Universal Single-Copy Orthologs
CO: CONSTANS
CRY1: CRYPTOCHROME 1
CYR2: CRYPTOCHROME 2
CjAG: Camellia japonica AGAMOUS homologous gene
cjaND: Camellia japonica “Naidong
FHY3: FAR-RED ELONGATED HYPOCOTYLS 3
FLC: FLOWERING LOCUS C
FT: FLOWERING LOCUS T
GI: GIGANTEA
Hi-C: High-resolution chromosome conformation capture analysis
Ks: Synonymous mutation rate
LG: Linkage group
SLAF-seq: Specific locus amplified fragment sequencing
TIR: Terminal inverted repeat
WGD: Whole-genome duplication event
WGCNA: Weighted gene co-expression network analysis
