# Author Correction: Genomics insights into flowering and floral pattern formation: regional duplication and seasonal pattern of gene expression in Camellia

**DOI:** 10.1186/s12915-024-01863-8

**Published:** 2024-03-12

**Authors:** Zhikang Hu, Zhengqi Fan, Sijia Li, Minyan Wang, Mingchuan Huang, Xianjin Ma, Weixin Liu, Yupeng Wang, Yifan Yu, Yaxuan Li, Yingkun Sun, Xinlei Li, Jiyuan Li, Hengfu Yin

**Affiliations:** 1 State Key Laboratory of Tree Genetics and Breeding, Research Institute of Subtropical Forestry, Chinese Academy of Forestry, Hangzhou, 311400 Zhejiang China; 2 College of Information Science and Technology, Nanjing Forestry University, Nanjing, 210037 China; 3 Key Laboratory of Forest Genetics and Breeding, Research Institute of Subtropical Forestry, Chinese Academy of Forestry, Hangzhou, 311400 Zhejiang China; 4 College of Horticulture, Qingdao Agricultural University, Qingdao, 266109 Shandong China

**Author Correction: BMC Biology 22 50, (2024)**

**https://doi.org/10.1186/s12915-024-01851-y**

In the original article [1], the respective images for Figs. 2 and 3 are erroneously transposed and should be treated as having been swapped over.
