# Supplementary material for: Genomics insights into flowering and floral pattern formation: regional duplication and seasonal pattern of gene expression in Camellia
Source: BMC Biol. 2024 Feb 27;22:50. doi: 10.1186/s12915-024-01851-y (PMC10900828; doi:10.1186/s12915-024-01851-y)
Supplement: Supplementary file 1 — Additional file 1: Fig. S1. The karyotyping and Kmer-based analyses of the cjaND genome. Fig. S2. The Hi-C heatmap shows the interaction of the chromosome. Fig. S3. The segregation patterns of the genetic makers. Fig. S4. The evolution and expression pattern of CjAGs. Fig. S5. Co-expression network analysis of CjAG1 and CjAG2. Fig. S6. Identification of annual rhythmic genes in C. japonica. Fig. S7. Identification of annual rhythmic genes in C. azalea. Fig. S8. The relationship of co-expression module of common rhythmic genes. Fig. S9. The seasonal expression genes participating in different pathways in C. japonica and C. azalea. Fig. S10. Identification of FT genes from C. japonica and C. azalea. [file 12915_2024_1851_MOESM1_ESM.docx]

**Additional File 1. Figures S1-S10.**

**
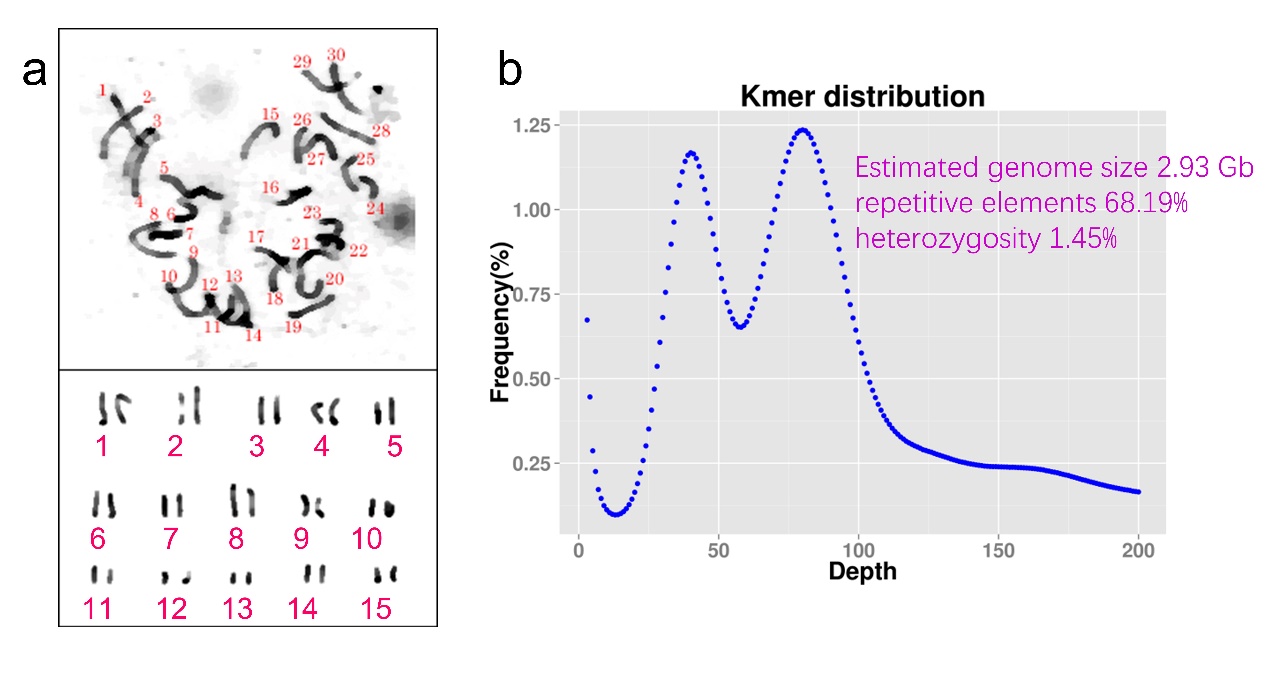
**

**Fig. S1 The karyotyping and Kmer-based analyses of the cjaND genome. a**, A karyotype image by the chromosome squash of young root-tips of the cjaND plant that have been used for genome sequencing (upper panel). The lower panel presents the chromosomes. **b**, The distribution of 21-bp Kmer of the cjaND genome. The Kmer abundance is used to calculate the estimated genome size, repetitive elements and heterozygosity.

**
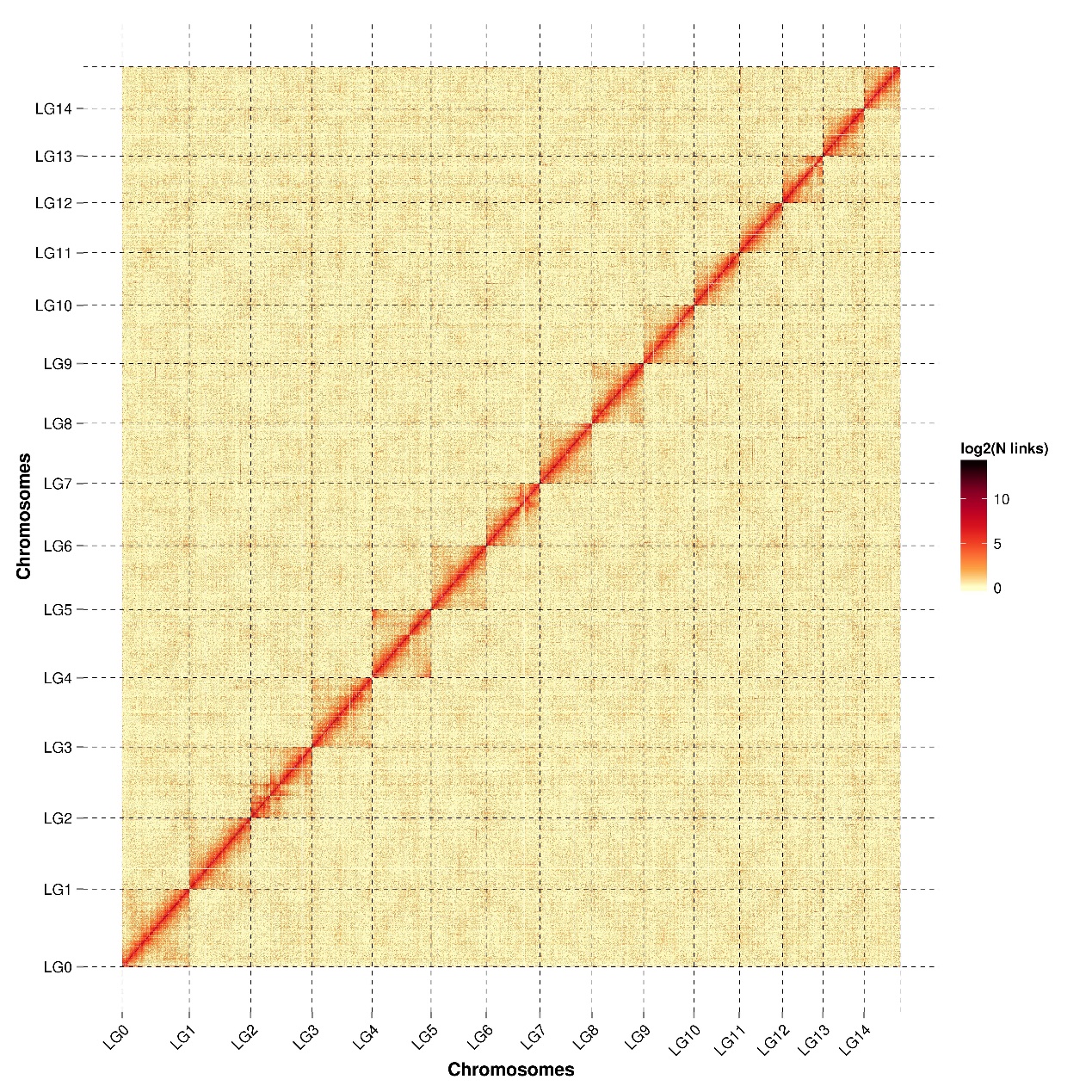
**

**Fig. S2 The Hi-C heatmap shows the interaction of the chromosome.** The horizontal and vertical coordinates represent the assembled chromosomes, and the color represents the interaction intensity between chromosomes, with the color from yellow to red representing the interaction intensity from small to large.

**
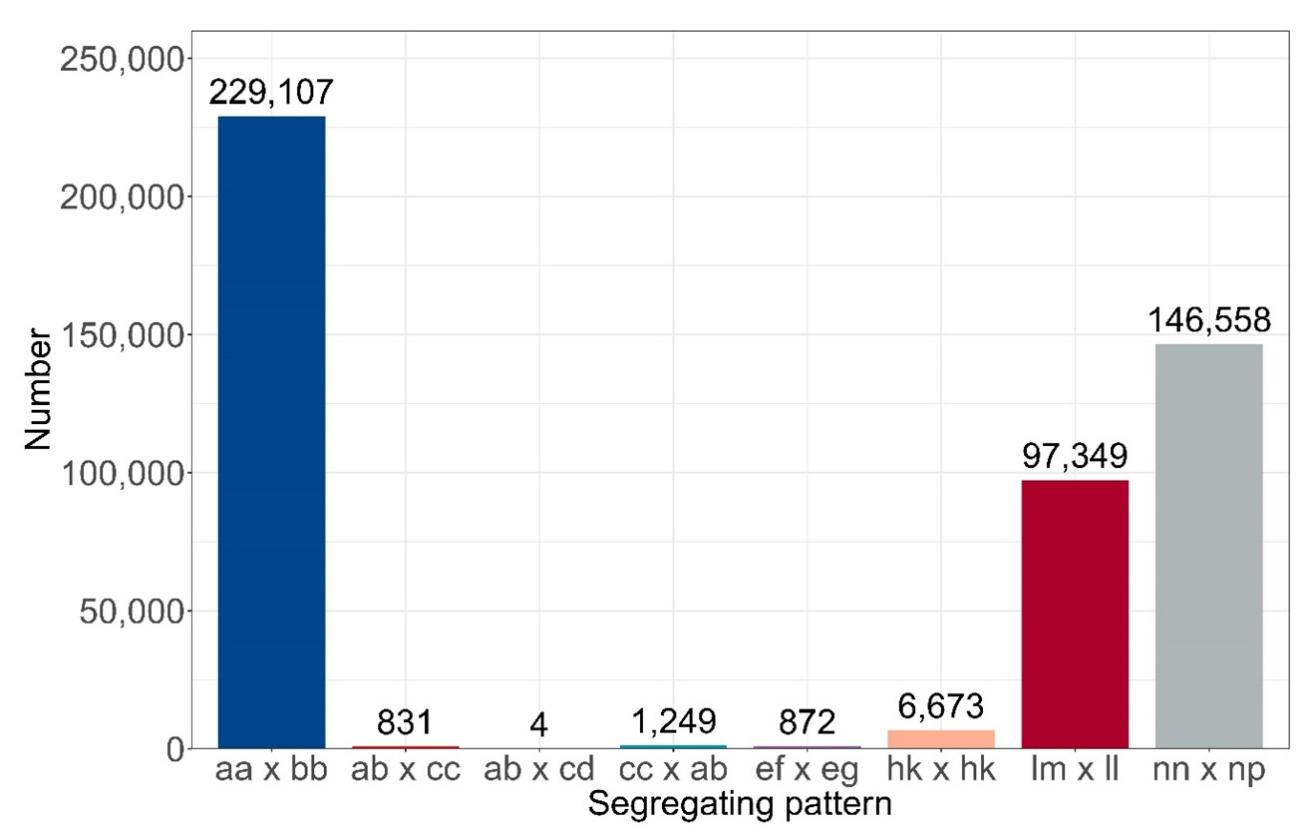
**

**Fig. S3 The segregation patterns of the genetic makers.** A cross-population of two closely-related *Camellia* species, *C. japonica* and *C. chekiangoleosa*, was used to construct a genetic linkage map. Distribution of each genetic segregation pattern. Segregating pattern indicates female parent genotype × male parent genotype in the SNP locus. Only the SNPs with the segregation patterns of ab × cc, ab × cd, cc × ab, ef × eg, hk × hk, lm × ll, and nn × np were used for genetic linage construction. and the vertical axis indicates the number of SNPs of this segregation pattern.

**
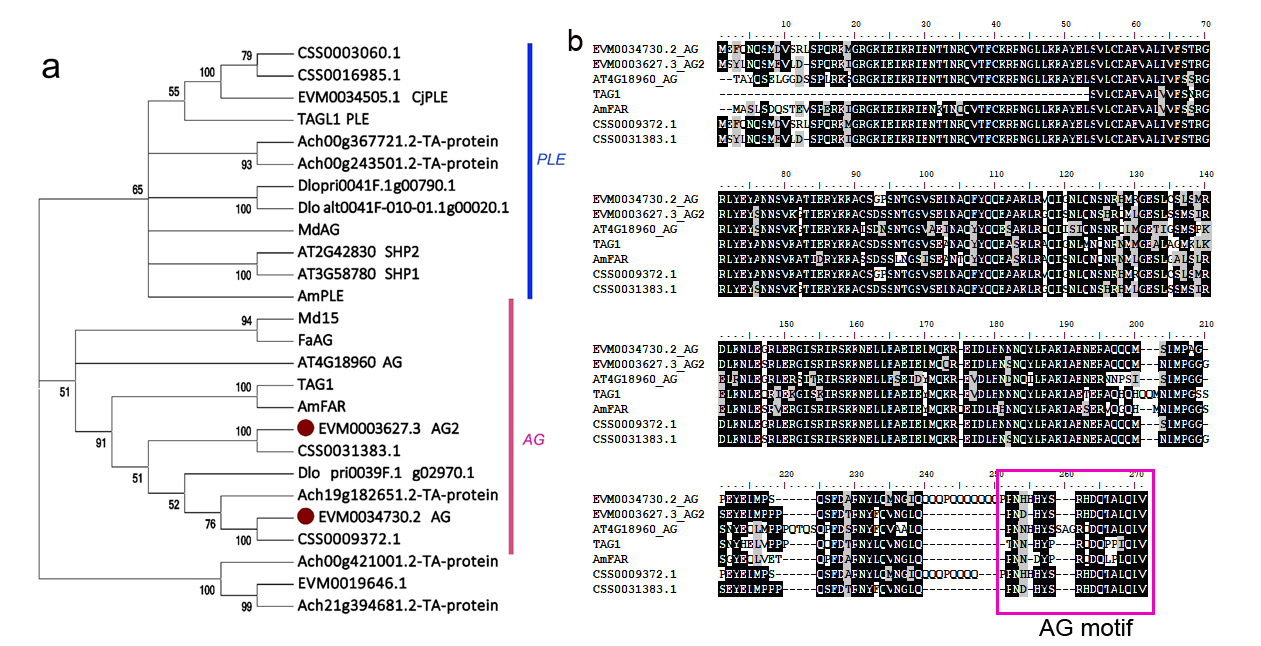
**

**Fig. S4 The evolution of *AGAMOUS* loci in *Camellia*. a,** A phylogenetic tree of C functional genes of *C. japonica* and closely-related species. The red dots represent the *AG* copies of *C. japonica*. The blue and red lines show the *PLE* lineage and *AG* lineage of C-class genes, respectively. **b,** Multiple sequence alignment of amino acid sequence of CjAGs with other known AGs. The red boxes show the conserved AG motif.

**
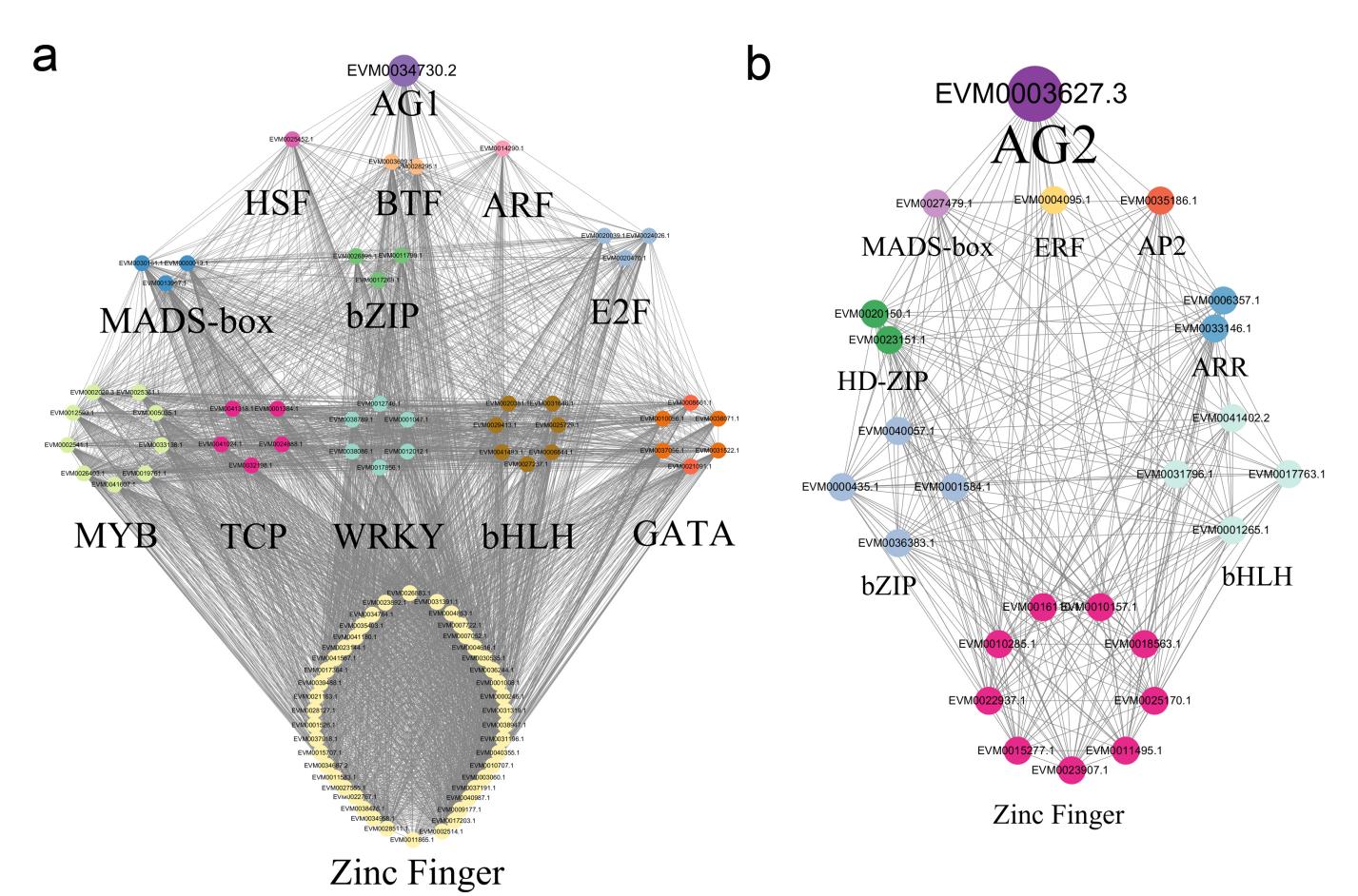
**

**Fig. S5 Co-expression network analysis of *CjAG1* and *CjAG2*.** The floral organ transcriptome containing 11 tissue types was obtained from single and formal double and anemone double flowers in *C. japonica* [6]. The WGCNA network was obtained and filtered to include transcription factors for visualization and interpretation for *CjAG1* (**a**, labelled as AG1 EVM0034730.2) and *CjAG2* (**b**, labelled as AG2 EVM0003627.3). The type of transcription factors are indicated by different colours.

**
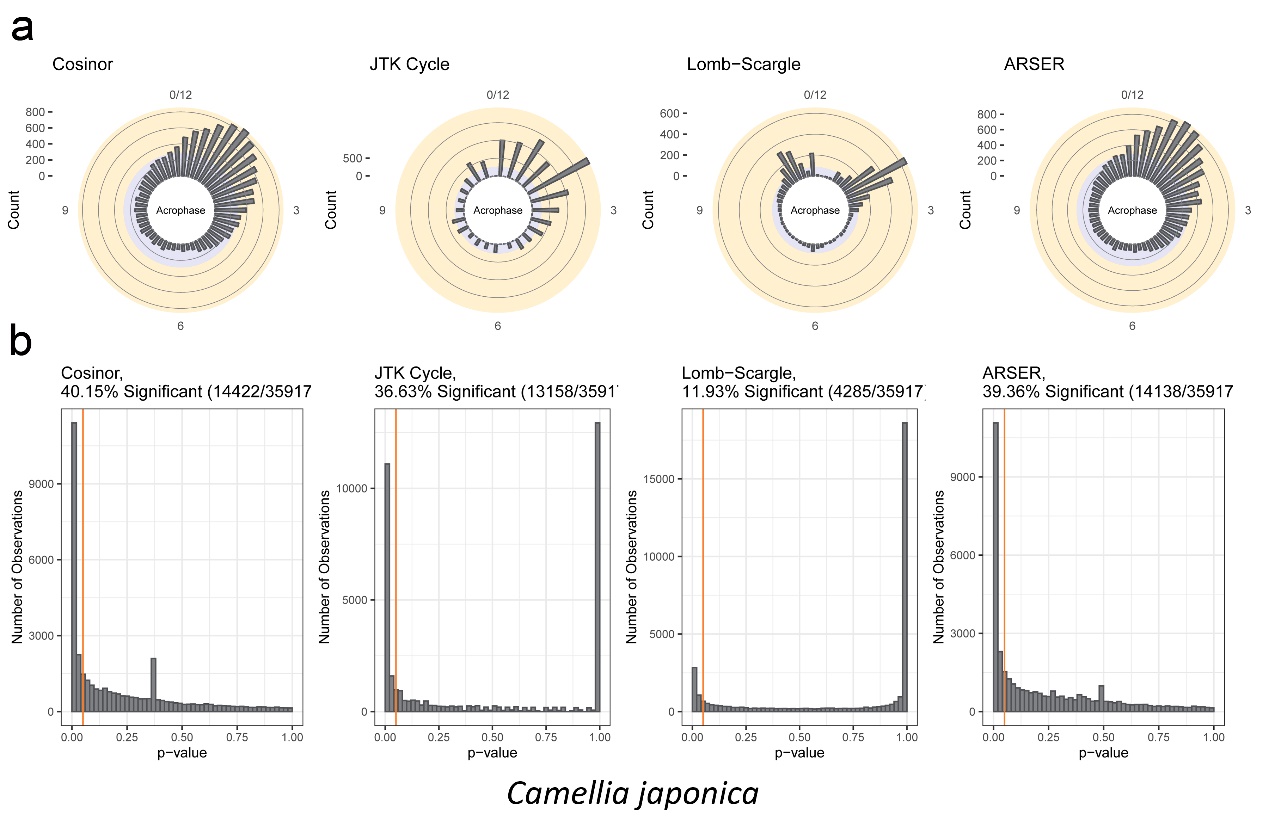
**

**Fig. S6 Identification of annual rhythmic genes in *C. japonica****.* **a**, The four modes including cosinor, JTK cycle, Lomb-Scargel and ARSER that are implemented in the R packaged DiscoRhythm are used to identify the annual rhythmic genes, and the acrophase of the prediction of genes in each model is revealed respectively (from left to right). **b**, The genes with significant correlation (p-value < 0.05) to the prediction models are identified as the candidate of rhythmic genes for the following analyses. The vertical orange line indicates the p-value cutoff of 0.05.

**
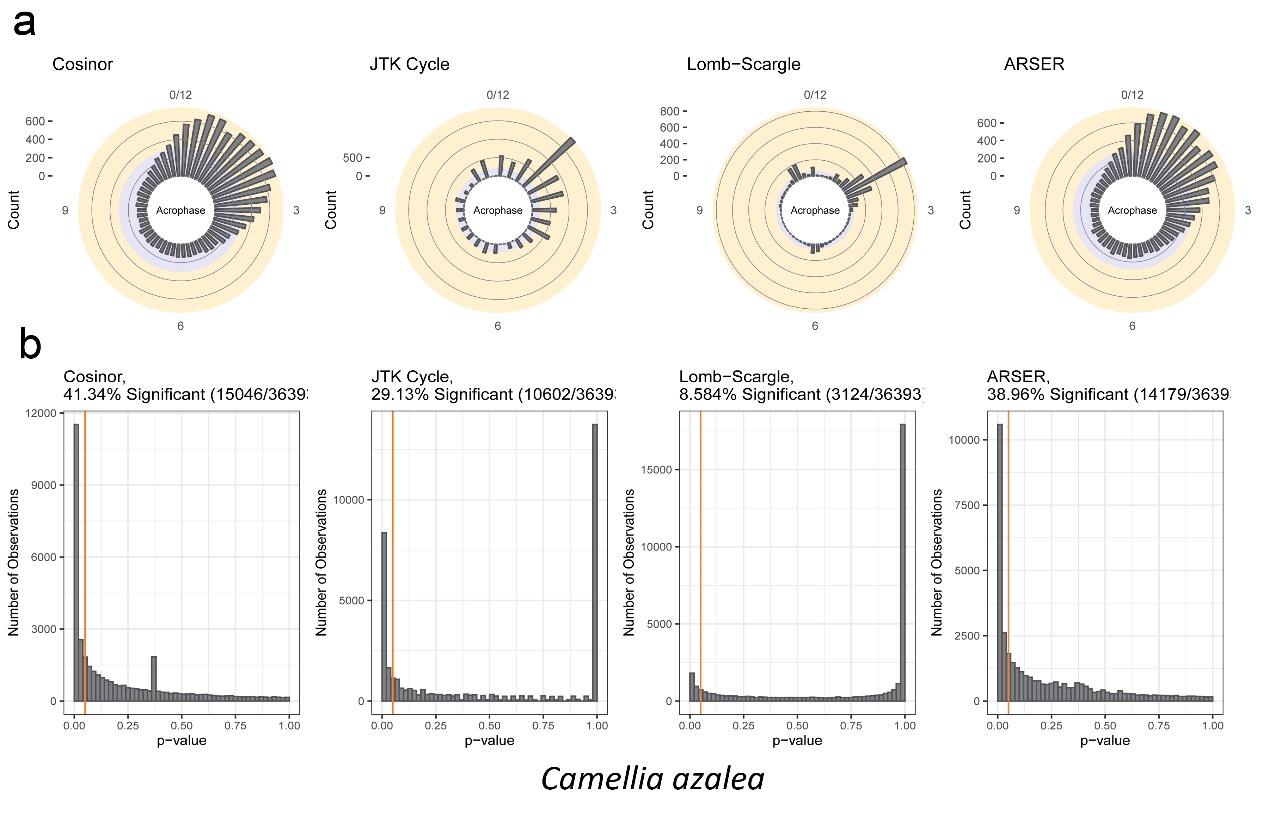
**

**Fig. S7 Identification of annual rhythmic genes in *C. azalea****.* **a**, **b** The identification and acrophase of the annual rhythmic genes as described in Fig. S6. The cjaND genome reference is used to obtain gene expression levels in *C. azalea*.

**
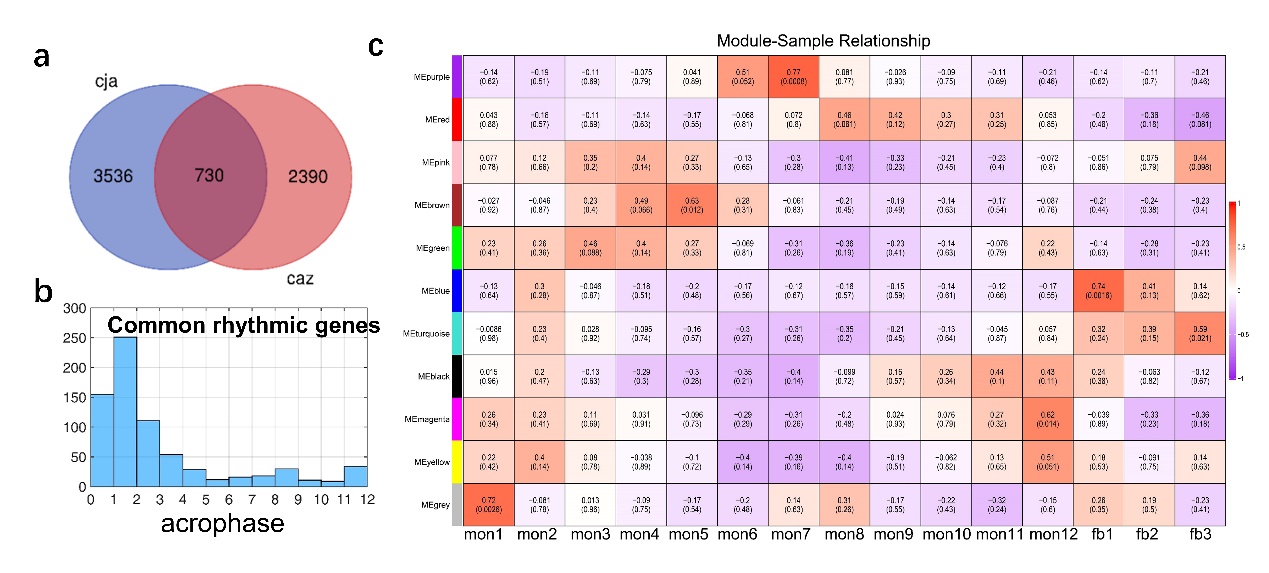
**

**Fig. S8 The relationship of co-expression module of common rhythmic genes. a,** The Venn diagram of rhythmic genes in *C. japonica* and *C. azalea.* The number in the blue circle represents the rhythmic genes of *C. japonica* and the red represents the rhythmic genes of *C. azalea*. **b,** Distribution of acrophase of the common rhythmic genes in each month. We used the 6656 rhythmic genes conducted the co-expression module. The relationship between module and sample was shown on right (**c**). In each box, the top number and color represent the correlation between module and sample, in which red represents a positive correlation, purple represents a negative correlation. The bottom number in each box represents the p-value of the Pearson’s correlation.

**
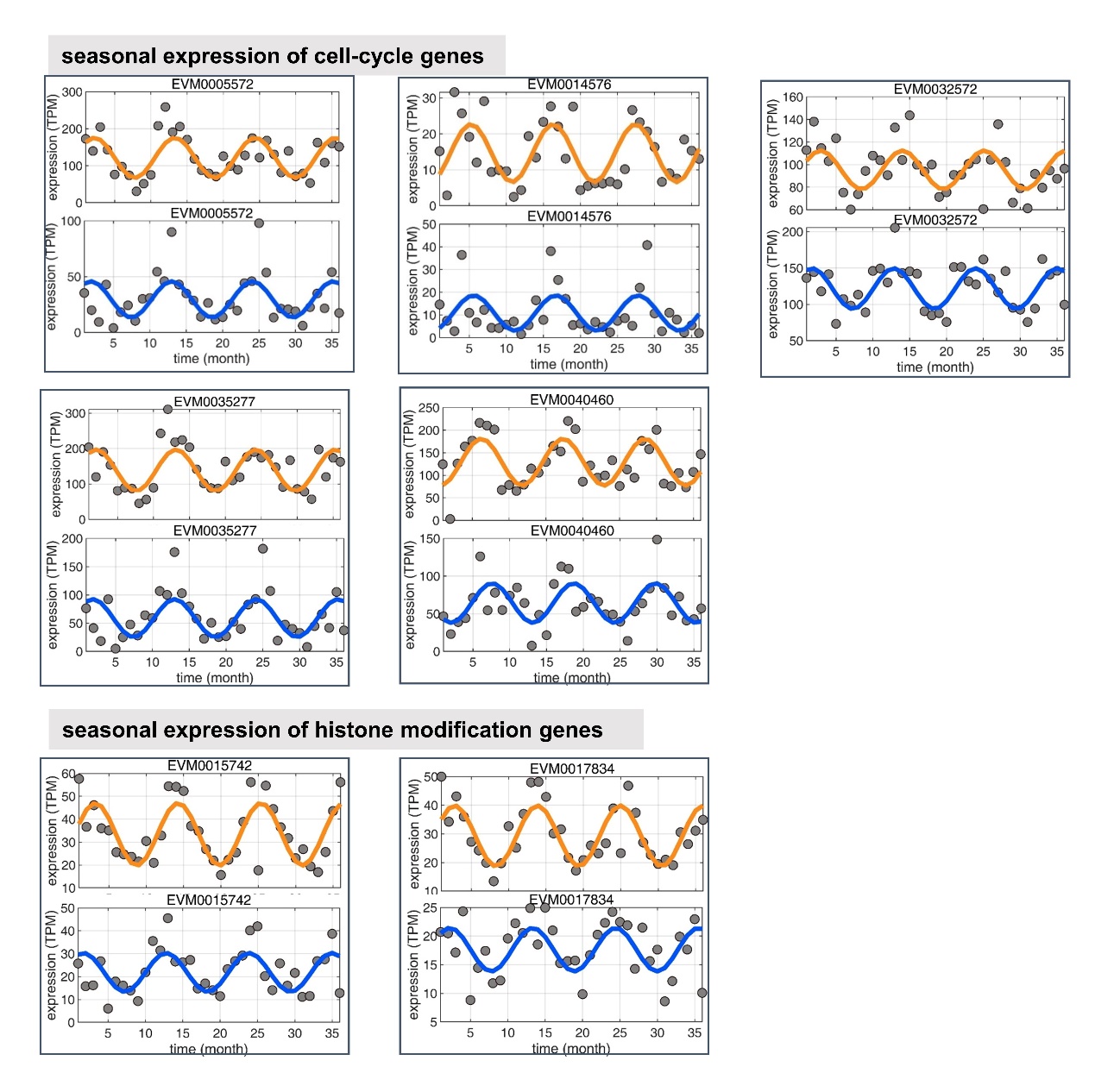
**

**Fig. S9 The seasonal expression genes participating in different pathways in *C. japonica* and *C. azalea*. a-e,** The seasonally expressed genes involved in the control of cell cycle. **f-g,** The seasonally expressed genes involved in the histone modification. The details of gene annotation are listed in Table S14. In each panel, the expression levels (TPM) of each candidate genes are scattered as grey dots, and orange (*C. japonica*) and blue (*C. azalea*) lines indicated the fitted rhythmic models.

**
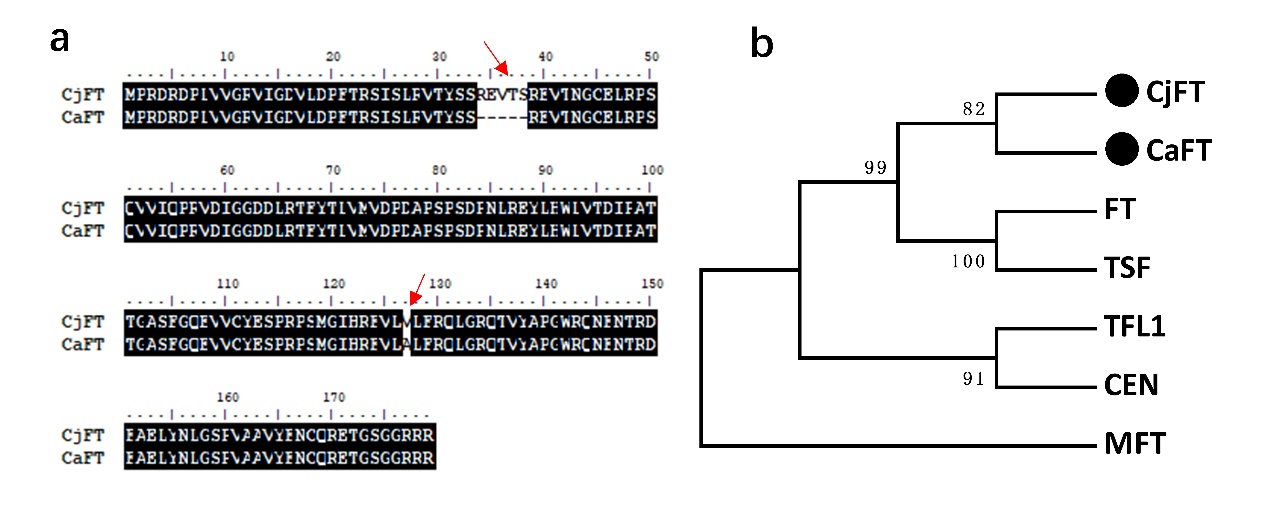
**

**Fig. S10 Identification of *FT* genes from *C. japonica* and *C. azalea*. a,** Alignment of protein sequences of CjFT and CaFT. Two locations of changes of amino acids are indicated by red arrows. **b,** Phylogenetic analysis of FT family genes from *C. japonica*, *C. azalea* and *Arabidopsis thaliana*. FT, AT1G65480; TFL1, AT5G03840; CEN, AT2G27550; MFT, AT1G18100; TSF, AT4G20370
